# Few-layer MoS_2_ dispersion induced by sulfur atom sharing to promote CO_2_ hydrogenation to methanol

**DOI:** 10.1039/d6ta01545j

**Published:** 2026-06-05

**Authors:** Gustavo A. S. Alves, Silvio Bellomi, Tobias Wagner, Alberto Tampieri, Christian Etzlstorfer, Michael Stöger-Pollach, Daniel C. Cano-Blanco, Karin Föttinger

**Affiliations:** a Institute of Materials Chemistry, TU Wien Getreidemarkt 9/BC/01 1060 Vienna Austria karin.foettinger@tuwien.ac.at; b University Service Center for Transmission Electron Microscopy (USTEM), TU Wien Stadionallee 2/057-02 1020 Vienna Austria; c Paul Scherrer Institute, PSI Center for Energy and Environmental Sciences Villigen PSI CH-5232 Switzerland; d École Polytechnique Fédérale de Lausanne (EPFL), Institute of Chemical and Bioengineering Lausanne CH-1015 Switzerland

## Abstract

With the ongoing interest in developing more stable and versatile catalysts for CO_2_ hydrogenation to methanol, molybdenum sulfide (MoS_2_) has been recently proposed as an alternative material. However, in its bulk state, CO_2_ hydrogenation over MoS_2_ typically favors methane formation. In this work, a wet impregnation method is applied for the production of ZnS-supported MoS_2_, as confirmed by characterization *via* X-ray Diffraction, Raman and X-ray Photoelectron Spectroscopy. In contrast with the negligible methanol production shown by the pure MoS_2_ reference, 2% MoS_2_/ZnS presents a methanol selectivity of 78% at a CO_2_ conversion of 2.3% under the mild reaction conditions of 200 °C and 20 bar. Density Functional Theory and Transmission Electron Microscopy suggest that the improved catalytic activity arises from an even dispersion of few-layer MoS_2_ with exposed basal plane sites at the ZnS surface, an arrangement possibly enabled by the structural similarity and the shared S atoms between 2H-MoS_2_ and W–ZnS phases. This hypothesis is strengthened by the comparison with the reference sample consisting of ZrO_2_-supported MoS_2_ sample, in which more agglomerated MoS_2_ particles resulted in a lower and less selective methanol production. Moreover, *in situ* X-ray absorption spectroscopy and H_2_ temperature-programmed reduction suggest further evidence of a MoS_2_/ZnS interaction during the H_2_ pretreatment, which may promote not only the expected formation of S-vacancies but also a partial reconstruction of MoS_2_ given the close contact and sharing of S atoms with the ZnS support.

## Abstract 2

With the ongoing interest in developing more stable and versatile catalysts for CO_2_ hydrogenation to methanol, molybdenum sulfide (MoS_2_) has been recently proposed as an alternative material.

## Graphical abstract

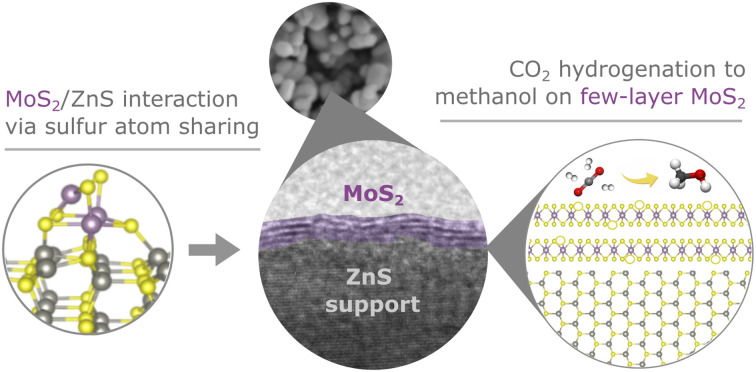

## Introduction

With the growing necessity of phasing out fossil fuels due to the concerning scenario of anthropogenic climate change, the introduction of alternative feedstocks for producing methanol could represent a significant step for a greener chemical industry, as the conventional production of this compound is currently responsible for about 10% of the total CO_2_ emissions in this growing sector.^1^ In this context, catalytic CO_2_ hydrogenation to methanol^2^ may play a key role in the near future, given the possibility of utilizing CO_2_ captured from air and a variety of point sources.^3^ Although Cu/ZnO/Al_2_O_3_ catalysts are considered the most well-known and established catalyst for this application,^4^ recent research suggests that alternative formulations including molybdenum sulfide (MoS_2_) may present comparable or even higher catalytic performance under similar reaction conditions.^5^ In contrast with Cu-based materials, the non-metallic nature of such catalysts may offer advantages in terms of stability and tolerance to sulfur impurities,^6^ which could benefit processes based on sulfur-rich CO_2_ from biogenic sources, for instance.^7^

In addition to CO_2_ hydrogenation, MoS_2_ has been already implemented as a catalyst for hydrodesulfurization^8^ and more recently researched as a photocatalyst^9^ or electrocatalyst^10^ for hydrogen production. Considering the layered structure of MoS_2_, these applications have been strongly associated with the reactivity of sulfur vacancies located at edge sites, while basal planes have been generally regarded as an inactive region.^11^ Similarly, in the context of CO_2_ conversion, MoS_2_ morphologies with abundant edges are correlated with a high activity for methane production.^12^ However, fewer edges and more extensive basal planes can discernibly shift the catalytic activity to favor the production of methanol instead of methane. For this reason, the most MoS_2_-based catalysts for CO_2_ hydrogenation reported up to date involve few-layer or single-layer MoS_2_, in which basal plane S-vacancies are considered the active site for methanol formation through a CO-based pathway.^5,13^ In a different manner, the introduction of other compounds in the MoS_2_ synthesis was also shown to be beneficial for methanol selectivity, as observed for ZnS^14^ as well as Na-,^15^ C-,^16^ Cu-,^17^ Ni-,^18^ Co-^19^ and Mn-based^20^ additives. While some of these promoters directly influence catalytic activity by tuning the content of S-vacancies and providing new sites for H_2_ activation, others exert an effect on the morphology of MoS_2_ in manner to prevent agglomeration and exposure of edge sites. As an example of the latter, the growth of MoS_2_ in spatial confinement with ZnS derived from a metal–organic framework was shown to create hollow composite nanocubes containing MoS_2_ with abundant basal-plane S-vacancies and high activity for CO_2_ hydrogenation to methanol.^14^

In this work, a facile wet impregnation method is applied to produce ZnS-supported MoS_2_. In comparison with bulk MoS_2_, the material shows superior catalytic performance for CO_2_ hydrogenation to methanol, as the strong interaction between the sulfides may inhibit agglomeration and promote the dispersion of few-layer MoS_2_ at the surface of the ZnS support.

## Experimental

### Catalyst synthesis

The synthesis precursor ammonium tetrathiomolybdate (ATTM) was obtained through a procedure similar to a previous report,^21^ in which H_2_S was bubbled into a mixture containing 80 g Na_2_MoO_4_·2H_2_O (Carl Roth, 99.5%) diluted in a concentrated ammonia aqueous solution. After about 30 minutes the mixture was cooled in an ice bath for the formation of a precipitate, which was later filtered and dried at room temperature.

ZnS-supported MoS_2_ has been synthesized by impregnating powder ZnS (Carl Roth, 99%) with an aqueous solution of ATTM. Initially, 2.5 g of ZnS are mixed with 50 mL H_2_O and the appropriate amount of ATTM according to the planned molar concentration of Mo (2 to 10% Mo) in the powder sample. Subsequently, the mixture is transferred to a rotary evaporator where it is dried at approximately 20 mbar. In a tubular furnace under N_2_ flow and a heating rate of 10 °C min^−1^, the solid is further dried at 120 °C for 1 h and finally thermally treated at 400 °C for 2.5 h. For comparison, bulk MoS_2_ was produced by thermal treatment of ATTM at the same conditions. Furthermore, ZrO_2_-supported MoS_2_ and C-supported MoS_2_ were obtained by an analogous method using ZrO_2_ powder (Fluka, 99%) or Carbon black (Printex XE 2 B, 99%) instead of ZnS.

### Characterization

The material has been analyzed by X-ray diffraction (XRD) in a Philips X'Pert diffractometer at 45 kV with a Cu Kα source, together with the Crystallography Open Database (COD)^22^ for further data interpretation. Furthermore, Raman spectroscopy was performed with a Bruker RAMANtouch microscope at 0.2 mW under excitation of a 532 nm laser. For the investigation of surface composition, X-ray Photoelectron Spectroscopy (XPS) was performed in a SPECS µ-Focus system (AlKα source, Phoibos 150 WAL detector). For XPS data analysis, the CasaXPS software^23^ was utilized and the C 1s peak related to adventitious carbon has been fixed at 284.8 eV for calibration of all spectra. Textural properties of the material were analyzed by N_2_ physisorption with a Micromeritics ASAP 2020, considering the Brunauer–Emmett–Teller (BET) method for surface area determination. The morphology of the samples was evaluated by Scanning Electron Microscopy (SEM) using a FEI Quanta 250FEG at 5 kV. Further structural features were analyzed by Transmission Electron Microscopy (TEM) with a FEI Tecnai F20 at an accelerating voltage of 200 kV.

*In situ* X-ray Absorption Spectroscopy (XAS) was performed at the B18 beamline of the Diamond Light Source. The experiments were conducted with powder samples mixed with boron nitride in a quartz capillary at 10 bar pressure and a total flow of 20 mL min^−1^. After purging with He, the cell was pressurized and heated at a 10 °C min^−1^ rate under H_2_ flow (10% H_2_ diluted in He) in order to simulate the catalyst pretreatment conditions at 300 °C during 1 h. Subsequently, the temperature was lowered to 200 °C and CO_2_ hydrogenation conditions were simulated by introducing CO_2_ in a 1CO_2_ : 3H_2_ ratio during 1 h, before cooling to room temperature. Mo K-edge XANES spectra were collected in transmission geometry, while gases released during the experiment were analyzed with a Pfeiffer OmniStar Mass Spectrometer. Processing and fitting of EXAFS data were performed using the Demeter software suite^24^ using *k* values between 3 and 9 and maintaining Δ*R* lower than 0.02 Å. Temperature-programmed reduction under H_2_ (H_2_-TPR) was performed with a Microtrac Belcat II, in which 80 mg of catalyst were pretreated under Ar flow at 300 °C, followed by cooling and heating 10 °C min^−1^ in a 5% H_2_ flow, while the outgas was measured by a Thermal Conductivity Detector (TCD). For Temperature-Programmed Desorption of H_2_ (H_2_-TPD) experiments, 70 mg of catalyst were pretreated at 300 °C under H_2_ atmosphere near atmospheric pressure, followed by cooling, evacuation to 0.01 mbar and heating at 10 °C min^−1^ while the desorbed gas was analyzed by a Pfeiffer OmniStar Mass Spectrometer. An analogous experiment was performed for CO_2_-TPD with the addition of a CO_2_ dosing step at 50 mbar for 15 minutes before evacuation to 0.01 mbar and heating.

### Catalyst testing

The catalytic activity of the samples was evaluated in the tubular fixed-bed reactor PID Effi from Micromeritics, under 1.25 mL_n_ min^−1^ CO_2_ + 3.75 mL_n_ min^−1^ H_2_ flow at 200 °C and 20 bar pressure. Shortly before reaction, a pretreatment with 5 mL_n_ min^−1^ H_2_ at 300 °C was performed for catalyst activation. The comparison of different MoS_2_ loadings was done with 1 g of catalyst and reaction products were detected in the gas phase with an Inficon Micro GC 3000 equipped with a Plot Q column and a TCD detector. Furthermore, experiments at higher Gas Hourly Space Velocity (GHSV) were performed with 0.5 g_cat_ and 0.25 g_cat_ using an Agilent 7890 GC with FID detection. For the comparison between 2% MoS_2_/ZnS and 2% MoS_2_/ZrO_2_, 0.25 g of sample was diluted with quartz sand to keep the catalyst bed volume constant. Except for the 165 h-long experiment, the reported product yields refer to approximately 18 h of time on stream.

### Density functional theory (DFT) simulation

DFT simulations were performed using the Vienna *Ab Initio* Simulation Package (VASP), version 6.3.^25^ The Perdew–Burke–Ernzerhof (PBE) generalized gradient approximation was employed using a plane-wave cutoff of 400 eV,^26^ and including non-spherical contributions of the gradient of density in the PAW spheres.^27,28^ The electronic convergence was eased using 0.05 eV width of Gaussian smearing. All the calculations included the long-range dispersion corrections as implemented in the zero-damping DFT-D3 approach.^29^ Following previous reports, ZnS was modelled with a reconstructed non-polar (100) termination composed of five layers, each of 12 Zn and 12 S atoms, generated from a cut of the bulk wurtzite structure (COD-ID 1011195). The two bottom layers were kept frozen in the bulk configuration during the optimization, while the uppermost three layers were allowed to relax.^30^ The ZrO_2_ surface was generated starting from the bulk monoclinic reference (COD-ID 1522143), and simulated using a (111) termination according to the literature. The slab was composed of four O–Zr–O tri-layers (each containing 16 Zr and 32 O atoms) of which only the two uppermost were allowed to relax during optimization.^31^ The interface of MoS_2_ with the supports was modelled using an Mo_3_S_6_ patch sliced from an ideal monolayer, including also a configuration with a missing S atom to explicitly describe the Mo–S under-coordination, which was let free to relax in all simulations. The choice of the Mo_3_S_6_ patch was motivated by the lattice mismatch between the MoS_2_ monolayer, ZnS (100), and ZrO_2_(111), which would require substantial artificial strain or prohibitively large supercells to construct coherent periodic overlayers (details in Table S1). The finite patch model therefore provides a computationally tractable and physically relevant approach to capture the local edge-mediated interactions, sulfur vacancy formation, and interfacial sulfur sharing. The initial magnetic moments were set as +1.0 µB for Mo, while all other atoms were initialized using 0 µB guesses. For all the models, 15 Å of vacuum were placed along the *z*-axis to avoid spurious period interactions. The relaxations were performed using a *Γ*-centered 2 × 2 × 1 Monkhorst–Pack grids,^32^ using thresholds for electronic energies and ionic forces of 10^−5^ eV and 0.05 eV Å^−1^, respectively. To improve the description of the electronic structure and obtain accurate energies, a finer 5 × 5 × 1 grid was used. The center of an occupied electronic band (*i*) was calculated from the projected density of states (PDOS) using the following equation, where *εD*_*i*_(*ε*) is the PDOS of the *i* orbitals weighted by the energy, *E*_F_ is the Fermi level, and *E*_0_ is the lower bound of the energy interval.1
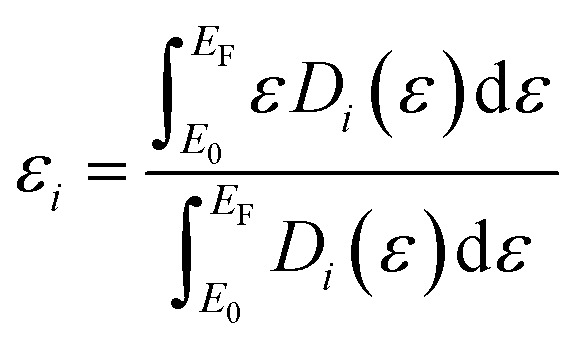


To quantify the similarity between two distinct PDOS *i* and *j*, the Tanimoto coefficient (*T*_C_),^33,34^ was used as defined in the following equation. *T*_C_ can be interpreted as the overlap between the areas spanned by the PDOS, and takes real values in the range [0,1], being equal to 0 (1) if the functions have no overlap (are identical).2
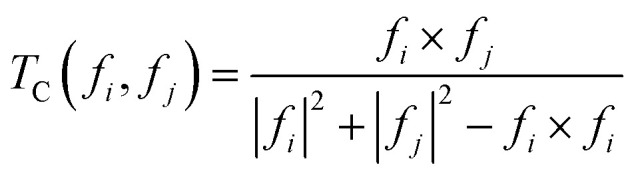


The thermodynamic penalty (Δ*E*_v_) for the introduction of a S vacancy in MoS_2_ was calculated for each support (S) using the following equation, as the energy difference between the most stable adsorption configurations of the pristine and defective patches, normalized by the number of MoS_2_ unit formula (*n*_MoS_2__ = 3).3
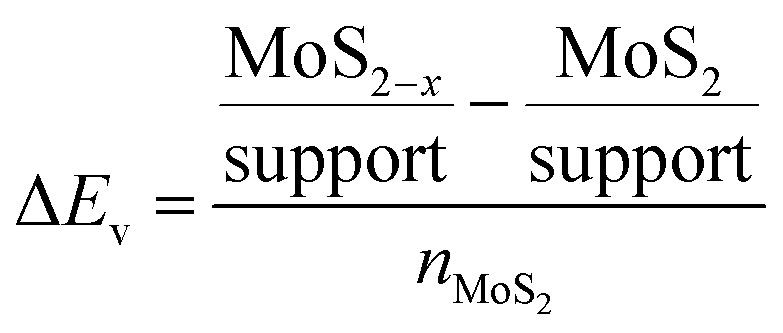


## Results and discussion

**Fig. 1:**
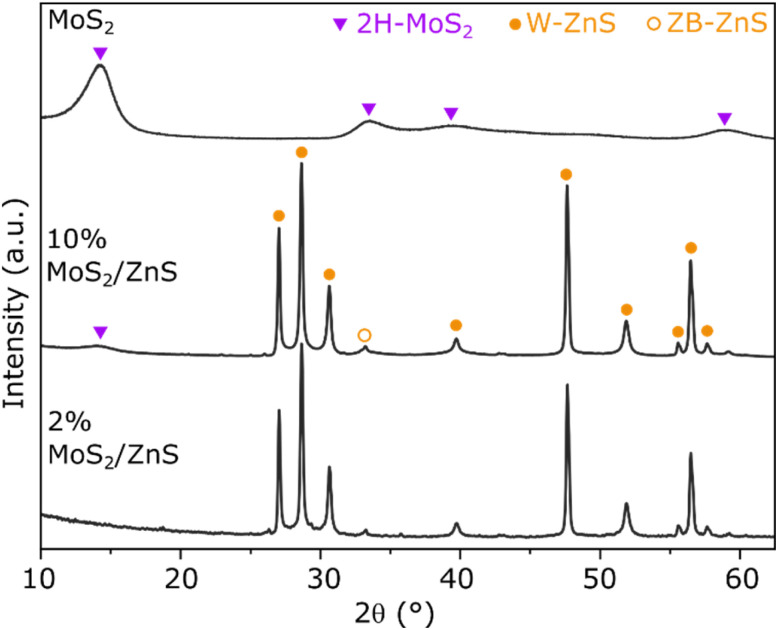
XRD patterns of bulk MoS_2_ and ZnS-supported MoS_2_ at 2% and 10% loading.

Bulk MoS_2_ and ZnS-supported MoS_2_ have been produced using the same procedure, ATTM as precursor and 400 °C annealing in inert atmosphere. In accordance with previous reports, bulk MoS_2_ produced by such a method consists of 2H-MoS_2_ (COD-ID 1010993) with low crystallinity, as evidenced by the wide XRD pattern in Fig. 1. The material presents a slab-like morphology with particle length varying from approximately 50 to 150 µm long, in addition to a N_2_ adsorption–desorption isotherm indicating mesoporous character with a BET surface area of about 35 m^2^ g^−1^, as shown by Fig. S1 and S2. After wet impregnation of ZnS with ATTM followed by thermal treatment, XRD confirms the formation of a similar 2H-MoS_2_ phase for the sample with 10% loading due the signal around 14°, the most intense reflection of bulk 2H-MoS_2_ attributed to the interlayer spacing of (002) planes. The remaining features are less intense and cannot be distinguished due to the relatively low loading and crystallinity of MoS_2_. In agreement, Fig. S3 shows that no features from 2H-MoS_2_ can be distinguished upon impregnation of ZnS with the lower loadings of 5%, 2% and 1%. In all samples containing the ZnS support, its XRD pattern consists of the hexagonal Wurtzite phase (W–ZnS, COD-ID 1011195) with a minor contribution of zinc blende (ZB–ZnS, COD-ID 1100043). Furthermore, no significant shifts and width changes are observed when comparing pure ZnS and the impregnated material in Fig. S3, which indicates that the crystalline structure of the support remains stable upon impregnation and thermal treatment.

**Fig. 2:**
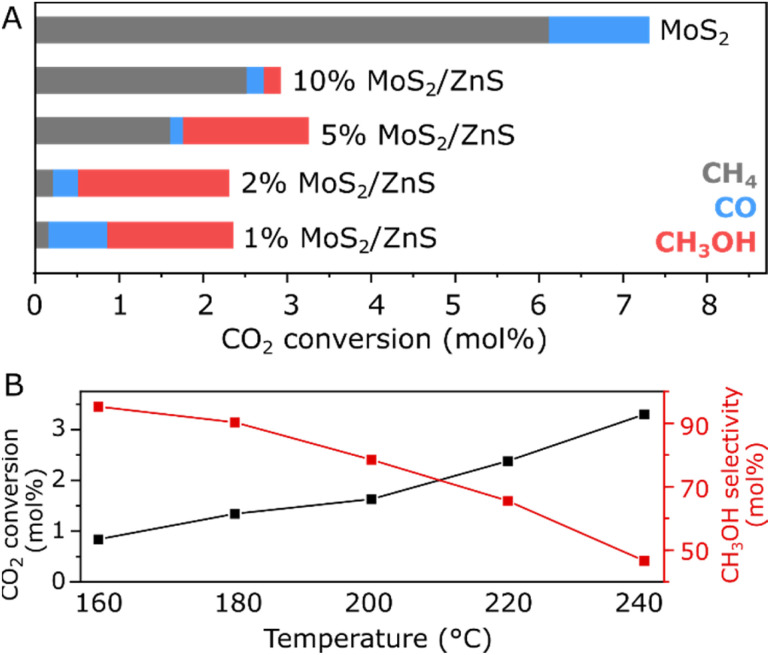
Catalytic activity of bulk MoS_2_ and ZnS-supported MoS_2_ in a fixed-bed reactor at 20 bar/200 °C/1 g_cat_ in terms of CO_2_ conversion to CH_4_, CO and CH_3_OH (A) and catalytic activity of 2% MoS_2_/ZnS at 20 bar/0.5 g_cat_ in terms of total CO_2_ conversion and selectivity to CH_3_OH at varied temperatures (B).

The catalytic activity of MoS_2_ and ZnS-supported MoS_2_ has been evaluated in a fixed bed reactor at 20 bar and 200 °C after a H_2_ pretreatment at 300 °C, previously suggested to optimally promote the activation of few-layer MoS_2_ catalysts through the formation of S-vacancies.^5,13^ Under these conditions, Fig. 2A shows that bulk MoS_2_ presents negligible methanol formation, as its total CO_2_ conversion of 7.3% corresponds mostly to CH_4_ with CO as a minor product. With the impregnation of 10% MoS_2_ on ZnS, the total CO_2_ conversion is sharply decreased to 2.9% while the high selectivity towards CH_4_ is maintained. This trend suggests that the catalytically active phase in 10% MoS_2_/ZnS may still be similar to bulk MoS_2_, coherently with the presence of agglomerated MoS_2_ with abundant edge S-vacancies, known to promote CO_2_ hydrogenation to methane.^5^ However, at lower MoS_2_ loadings a distinct trend is observed as the CH_4_ yield progressively decreases and CO_2_ hydrogenation to methanol becomes considerably more prominent with the selectivity of 46, 78 and 64% while comparable CO_2_ conversions of 3.2, 2.3 and 2.3% are maintained upon the MoS_2_ loadings of 5, 2 and 1%, respectively. The favorable catalytic activity at low MoS_2_ loading suggests a synergistic interaction between MoS_2_ and the support, given that pure ZnS presents negligible activity for CO_2_ hydrogenation.^14^ For further insights on the kinetic performance of 2% MoS_2_/ZnS, catalytic testing has been conducted in a 165 h-long experiment at varying temperatures, complemented by a comparison of different GHSV values as shown in Fig. S4 and S5, respectively. As summarized in Fig. 2B, lower reaction temperatures promote higher CH_3_OH selectivity at the expense of lower CO_2_ conversions. In fact, between 160 °C and 240 °C, selectivity to methanol drops from 95 to 47% while total CO_2_ conversion increases from 0.8 to 3.3%. Interestingly, returning to the reaction temperature of 200 °C after the 240 °C step leads to methanol yields approximately 50% higher than the initial values, while CO and CH_4_ production rates remain similar, as shown in Fig. S4. Such an improvement could be associated to extensive formation of surface S-vacancies due to the long reaction time, as suggested by previous studies on other MoS_2_-based catalysts,^5,13,15^ although the 240 °C step may play a role on accelerating such process. In view of the promising catalytic activity of 2% MoS_2_/ZnS at the mild reaction conditions of 20 bar and 200 °C, the as-synthesized material was further characterized, seeking to better understand the nature of its active phase and role of the H_2_ pretreatment on the catalyst structure.

As the low content of the active phase in 2% MoS_2_/ZnS prevents clear detection *via* XRD, Raman spectroscopy has been employed, given its sensitivity to detect vibrational modes of layered structures from monolayer to multilayer stacking.^35^

**Fig. 3:**
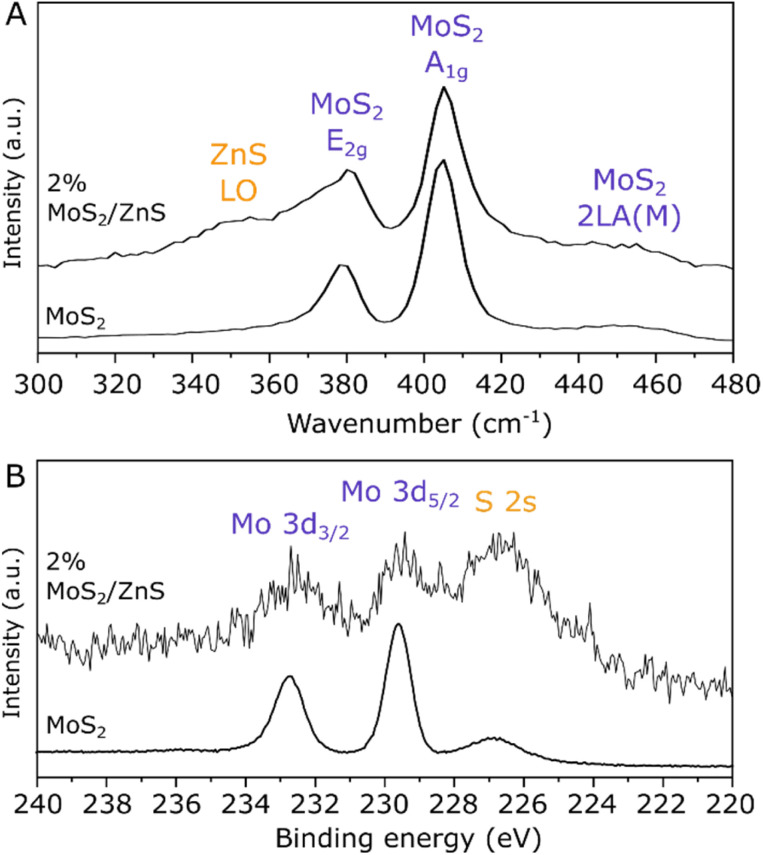
Raman spectrum with 532 nm excitation (A) and high-resolution XPS spectrum of the Mo 3d/S 2s region (B) of pure MoS_2_ and 2% MoS_2_/ZnS.

Accordingly, the spectrum of 2% MoS_2_/ZnS in Fig. 3A contains the LO mode from ZnS at 351 cm^−1^ (ref. 36) in addition to bands related to E_2g_, A_1g_ and 2LA modes from MoS_2_ at 380, 405 and 453 cm^−1^,^35^ respectively. As an indicator of MoS_2_ layer stacking, a difference of 25 ± 2 cm^−1^ is observed between E_2g_ and A_1g_ in both samples, consistently with stacking of at least 4 layers.^35^ Furthermore, analysis of the catalyst surface by XPS in Fig. 3B provides additional evidence for Mo species, as the Mo 3d doublet can be clearly distinguished from the S 2s peak at 226.5 eV, considering the characteristic doublet separation of 3.1 eV.^37^ While 2% MoS_2_/ZnS presents a low-intensity Mo 3d doublet with respect to the S 2s feature due to its low Mo surface content, Fig. S6 shows that the material loaded with 10% MoS_2_ has a similar XPS profile to that of pure MoS_2_, coherently with the higher prominence of MoS_2_ in the XRD pattern of this sample. Importantly, in all cases the presence of MoS_2_ at the catalyst surface is consistent with the binding energy of Mo 3d_5/2_ at 229.6 eV, which indicates a Mo^4+^ oxidation state and rules out surface oxidation of the sulfide in contact with air due the absence of Mo^6+^ species.^37,38^

**Fig. 4:**
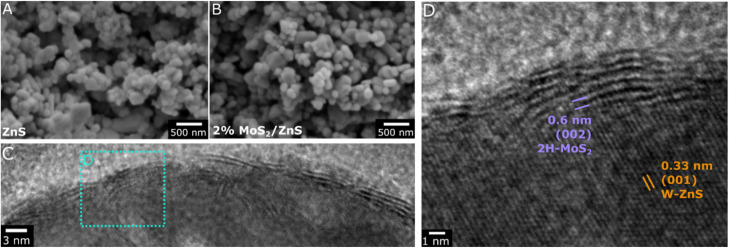
SEM micrographs of ZnS before and after impregnation with 2% MoS_2_ (A and B) and TEM images of 2%MoS2/ZnS (C and D).

Although Raman and XPS confirm the impregnation of MoS_2_ on ZnS, the SEM micrographs of the pristine support and the one impregnated with 2% MoS_2_ in Fig. 4A and B present an identical morphology, consisting of similarly rounded particles with smooth surfaces and approximately 100 to 400 nm in size. This agrees with the high dispersion observed from XRD and the homogenous stacking of few layers confirmed by Raman. Accordingly, the material impregnated with 10% MoS_2_ shows clearly segregated particles much larger than those attributed to ZnS, as additional evidence for bulk MoS_2_ upon such a high catalyst loading. As shown in Fig. S2, the negligible porosity of the ZnS support is maintained after MoS_2_ impregnation while its surface area decreases from 9.3 to 6.7 m^2^ g^−1^ at 2% loading and 6.3 m^2^ g^−1^ at 10% loading. Furthermore, TEM analysis has been performed to obtain further insights into the structure and morphology of the dispersed MoS_2_ phase. As shown in Fig. 4C and S7, the ZnS particles are predominantly covered by a shell with a distinctive crystalline structure, consisting of an arrangement of 3 to 7 stacked planes extending longitudinally along the rounded support surface. In fact, the magnified image in Fig. 4D reveals that these planes are separated by approximately 0.6 nm, which is the characteristic interplanar spacing of (002) layers in the 2H-MoS_2_ phase. These results suggest that MoS_2_ interacts with the ZnS support preferentially through basal planes, possibly due to the shared S atoms and the similar hexagonal structures of 2H-MoS_2_ and W–ZnS,^39^ thus leading to the growth of thin and extensive MoS_2_ layers in parallel to the ZnS surface, rather than in segregated MoS_2_ particles. Such a morphology may be a key factor to explain the catalytic activity of 2% MoS_2_/ZnS, as the exposed basal planes in few-layer MoS_2_ are expected to provide active sites for CO_2_ hydrogenation to methanol.^5^ Furthermore, the curved shape of the MoS_2_ layers might also play a contributing role, as the presence of structurally strained layers has been previously associated with higher activity for methanol production.^17,40^

In order to better understand the interaction between the active phase and the support in 2% MoS_2_/ZnS, the catalyst has been compared with pure MoS_2_ in an *in situ* XAS experiment under reaction-relevant conditions, consisting of a catalyst activation step at 300 °C under H_2_ followed by exposure to a CO_2_ + 3H_2_ mixture at 200 °C. As expected, the H_2_ pretreatment promotes the formation of sulfur vacancies with the release of H_2_S, which has been detected for both samples *via* mass spectrometry, as shown in Fig. S8. During the subsequent step, no methanol has been detected as a reaction product, given that the experiment could only be performed with approximately 5 mg of catalyst at the pressure of 10 bar.

**Fig. 5:**
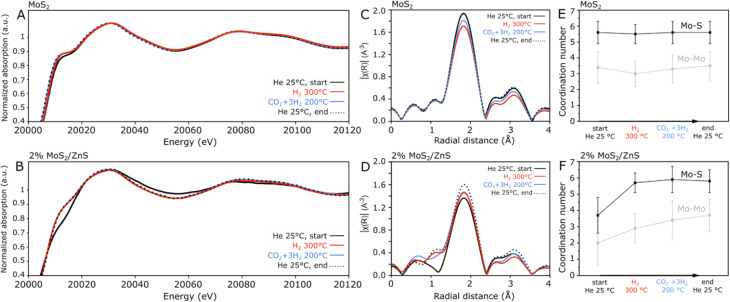
*In situ* Mo K-edge XANES spectra with the respective EXAFS and EXAFS fitting summary for MoS_2_ (A, C, E) and 2% MoS_2_/ZnS (B, D, F), collected at 10 bar.

In the XANES spectra shown in Fig. 5A, the Mo K-edge of MoS_2_ presents the typical features associated with the 2H-MoS_2_ phase,^41^ and despite the detection of H_2_S during the H_2_ pretreatment, only negligible changes occur throughout the *in situ* experiment. This suggests that the formation of S-vacancies was not pronounced enough to elicit a deep structural modification detectable by XAS, but rather limited to the surface. In contrast with the high stability of bulk MoS_2_, considerable differences are observed for 2% MoS_2_/ZnS in Fig. 4B, as the fresh catalyst has a considerably lower intensity in the 20 015 eV region. Furthermore, during the H_2_ treatment, the spectrum undergoes considerable modification, and these changes persisted during reaction-like conditions and subsequent cooling to room temperature. In view of the XANES data, EXAFS analysis in Fig. 5C and D provides a more detailed description in terms of Mo–S at and Mo–Mo coordination spheres, associated to the features at approximately 1.8 and 3.1 Å, respectively. While in pure MoS_2_ the intensity changes of EXAFS profiles can be simply associated to sample temperature due to its effect on the Debye–Waller factor,^42^ 2% MoS_2_/ZnS presents a more complex trend, with the H_2_ treatment promoting a clear increase in the Mo–S peak rather than the decrease observed for MoS_2_.

For a more comprehensive interpretation of such patterns, EXAFS fitting has been performed, using Mo–S and Mo–Mo paths of 2.41 and 3.15 Å, based on the 2H-MoS_2_ reference (COD-ID 1010993).^43^ Considering the results presented in detail in Fig. S9 and Table S2, a summary of the fitted coordination numbers during the *in situ* experiment is shown in Fig. 5E and F. Although the ideal 2H-MoS_2_ would show the same Mo–S and Mo–Mo coordination number of 6, the approximate values of 5.6 ± 0.7 and 3.4 ± 1.0 have been fitted for the bulk MoS_2_ sample. The relatively low Mo–Mo coordination number can be associated with the limited crystallinity of the material,^41^ coherently with the broad reflections verified in XRD analysis. In line with the XANES and EXAFS results, merely negligible changes are visible in the MoS_2_ fitting during the subsequent steps of the *in situ* experiment. On the other hand, a more pronounced difference is observed for 2% MoS_2_/ZnS (Fig. 5F), with much lower initial coordination numbers of 3.7 ± 1.1 and 2.0 ± 1.4 for Mo–S and Mo–Mo, respectively. This suggests that in the as-synthesized material, highly defective MoS_2_ interacts with the ZnS support, which rearranges following the H_2_ treatment at 300 °C, as reflected by the clear increase in the Mo–S coordination, accompanied by an increment in Mo–Mo. Following minor changes at reaction-similar conditions and cooling to room temperature, the spent 2% MoS_2_/ZnS sample presents Mo–S and Mo–Mo coordination numbers comparable to those of pure MoS_2_. Correspondingly with the XRD, XPS and SEM characterization, 10% MoS_2_/ZnS shows XANES and EXAFS profiles seemingly in between those of 2% MoS_2_/ZnS and MoS_2_, as the material consists of a combination of bulk and ZnS-supported MoS_2_.

According to the characterization of 2% MoS_2_/ZnS, the initially defective MoS_2_ shell is in close contact with the ZnS support, and the interaction between the sulfides promotes a partial restructuring of MoS_2_ during the H_2_ pretreatment, possibly due to filling of S-vacancies with sulfur derived from the ZnS support at the MoS_2_/ZnS interface. Nevertheless, the increase in Mo–S coordination does not rule out the formation of sulfur vacancies at the catalyst surface, as simultaneous H_2_S formation is verified by mass spectrometry as shown in Fig. S8. Furthermore, the few-layer character of the active phase is not altered during the H_2_ pretreatment, as TEM analysis in Fig. S10 shows a similar morphology to the as-synthesized material.

**Fig. 6:**
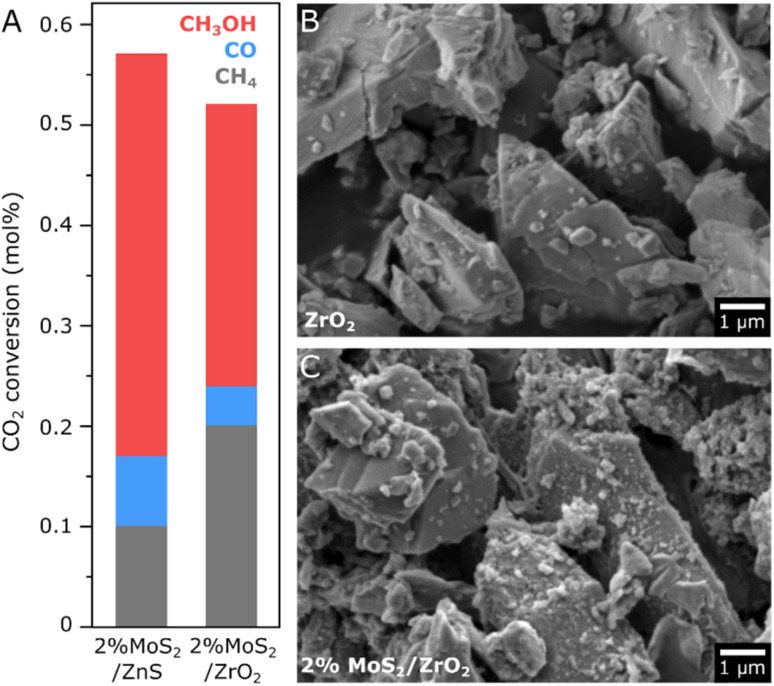
Catalytic activity of 2% MoS_2_/ZnS and 2% MoS_2_/ZrO_2_ at 20 bar/200 °C/0.25 g_cat_ in terms of CO_2_ conversion to CH_4_, CO and CH_3_OH (A) and SEM micrographs of ZrO_2_ before and after impregnation with 2% MoS_2_ (B) and (C).

With the observation of few-layer MoS_2_ at the surface of ZnS and the subsequent catalytic activity for CO_2_ hydrogenation to methanol, a comparison with a ZrO_2_ reference support has been performed in order to better understand the influence of the support material on the active phase, given the typical application of ZrO_2_ as a catalyst support for CO_2_ hydrogenation.^44,45^ After the synthesis of 2% MoS_2_/ZrO_2_ by an analogous impregnation method, the formation of MoS_2_ has been confirmed by the characteristic E_2g_ and A_1g_ vibrational bands in Raman spectroscopy and the prominent Mo^4+^ features in the XPS spectrum, as shown in Fig. S11. In a catalytic activity test at 200 °C summarized in Fig. 6A, 2% MoS_2_/ZrO_2_ exhibits a methanol yield corresponding to 70% of the value obtained for 2% MoS_2_/ZnS, while methane production is doubled. Unlike the well-dispersed impregnation of MoS_2_ on ZnS, SEM micrographs of 2% MoS_2_/ZrO_2_ in Fig. 6A and B show a much rougher surface morphology in comparison with the pure ZrO_2_ support. Such a difference in dispersion suggests that the interaction of MoS_2_ with the ZrO_2_ surface does not favor homogeneous growth of a layered structure as in ZnS, thus leading to extensive agglomeration of MoS_2_ in the form of nanoparticles with abundant edge sites, which may explain the less favorable selectivity for methanol production in comparison to the ZnS support. In accordance with the rougher morphology observed after impregnation, the BET surface area of 2% MoS_2_/ZrO_2_ is 3.7 m^2^ g^−1^, slightly higher than the pure support at 2.6 m^2^ g^−1^, while a similarly low porosity is verified in both N_2_ isotherms. Furthermore, in view of recently reported carbon-based supports for highly active MoS_2_ catalysts,^40^ a 2% MoS_2_/C catalyst has been additionally synthesized. In a preliminary comparison with 2% MoS_2_/ZnS, the material presents a lower selectivity of about 34%, despite the doubled methanol yield, as shown in Fig. S12.

**Fig. 7:**
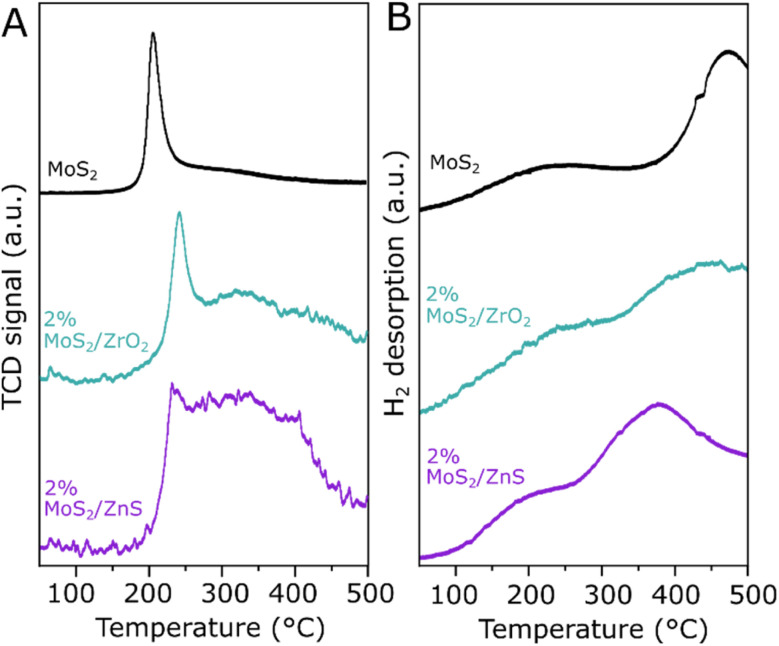
H_2_-TPR profiles after a 300 °C pretreatment in Ar (A) and H_2_-TPD profiles after a 300 °C pretreatment in H_2_ (B) for MoS_2_, 2% MoS_2_/ZrO_2_ and 2% MoS_2_/ZnS.

In order to better understand the interaction of CO_2_ and H_2_ with the surface of 2% MoS_2_/ZnS in comparison with bulk and ZrO_2_-supported MoS_2_, temperature-programmed desorption (TPD) and reduction (TPR) experiments have been performed. In the CO_2_-TPD results shown in Fig. S13, CO_2_ is desorbed from bulk MoS_2_ between 300 and 500 °C, indicating the presence of moderate to strong basic sites. Both 2% MoS_2_/ZnS and 2% MoS_2_/ZrO_2_ show a less intense but similar desorption profile, which suggests similar basicity to bulk MoS_2_. Despite such apparent similarity in CO_2_ interaction, the samples exhibit considerable differences in H_2_-TPR and H_2_-TPD experiments, as shown in Fig. 7. In line with previous reports, Fig. 7A shows a sharp reduction feature for MoS_2_ around 205 °C, associated with the release of H_2_S and the simultaneous formation of sulfur vacancies. In the supported MoS_2_ samples, this peak is shifted to approximately 235 °C while a broader reduction feature arises in the range between 250 and 450 °C, the latter being particularly prominent for 2% MoS_2_/ZnS. Considering that the pure ZrO_2_ and ZnS supports do not show any significant reduction within the investigated temperature range, as shown in Fig. S13, this additional feature can be attributed to the MoS_2_-support interface. Especially in 2% MoS_2_/ZnS, the additional reduction feature above 250 °C may provide further evidence for the MoS_2_/ZnS interaction suggested by *in situ* XAS experiment conducted under H_2_ at 300 °C, as the dispersion of defective MoS_2_ at the ZnS surface may help extracting S atoms from the support at the MoS_2_/ZnS interface during catalyst pretreatment. In Fig. 7B, the H_2_ desorption profile of MoS_2_ shows two distinct features at approximately 220 °C and 475 °C. While the region at low temperature remains similar in the supported samples, the high-temperature H_2_ desorption peak is shifted to 440 °C for 2% MoS_2_/ZrO_2_ and 375 °C for 2% MoS_2_/ZnS. Such changes suggest that H_2_ adsorbs less strongly on the supported MoS_2_ catalysts than the bulk MoS_2_. The trend correlates with the catalytic activity, where bulk-like MoS_2_ converted more CO_2_ to CH_4_ than MeOH, suggesting that high surface coverage of hydrogen could promote such over-reduction.

**Fig. 8:**
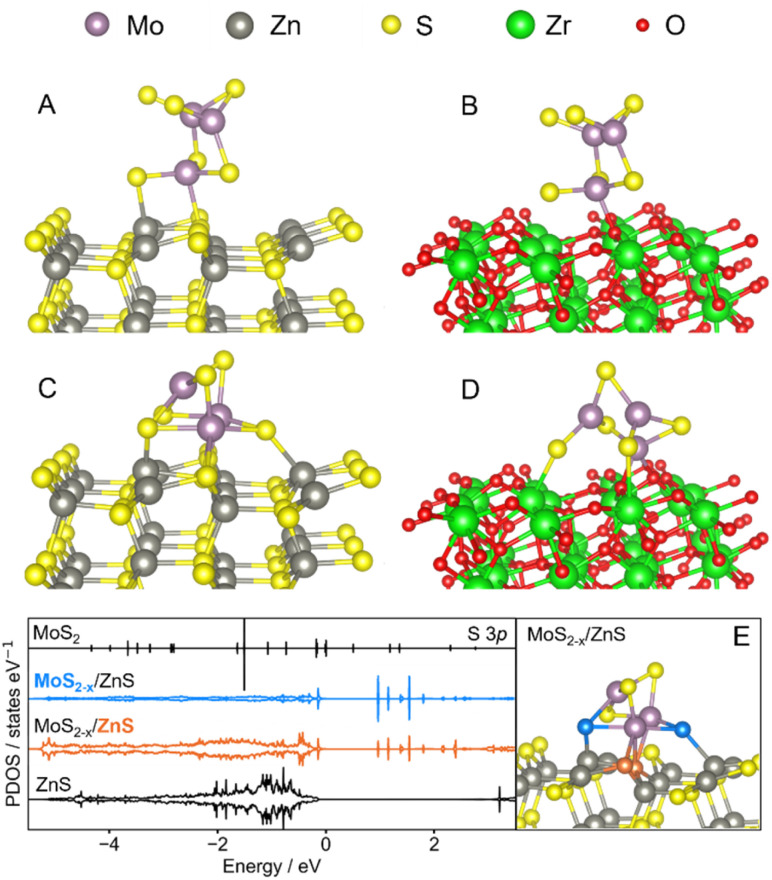
Side view of the optimized interfaces: MoS_2_/ZnS (A), MoS_2_/ZrO_2_ (B), MoS_2−*x*_/ZnS (C), MoS_2−*x*_/ZrO_2_ (D) and MoS_2−*x*_/ZnS interface highlighting the shared S atoms (coloured blue and orange) and their corresponding density of states projected on the S 3p states, together with MoS_2_ and ZnS references (E).

To rationalize the support-dependent MoS_2_ dispersion and restructuring observed experimentally, the interaction of pristine and sulfur vacant MoS_2_ patches was studied in DFT simulations on the non-polar ZnS (100) and ZrO_2_ (111) terminations, shown in Fig. S15, using thermodynamic metrics and the analysis of the projected density of states (PDOS).^30,31,46^ The patch model avoids the large artificial strain associated with constructing commensurate MoS_2_ overlayers on the lattice-mismatched ZnS(100) and ZrO_2_(111) surfaces, while capturing the local interfacial interactions. As shown by the optimized structural models in Fig. 8A and B, pristine MoS_2_ is primarily stabilized through interactions at under-coordinated edge sites on both supports, although a qualitative difference emerges upon formation of a sulfur vacancy. On ZnS, Fig. 8C shows that a reconstruction in which sulfurs are shared across the interface is the most stable configuration: sulfur atoms from ZnS coordinate to Mo edge sites while sulfur atoms originally belonging to MoS_2_ bind to Zn. Interestingly, even in presence of an S-vacancy the characteristic geometry of the MoS_2_ patch is retained at the interface with ZnS. This correlates with the favorable growth of homogeneous MoS_2_ layers in close contact with the ZnS surface observed *via* TEM. Furthermore, such a pattern is not observed for the ZrO_2_-based model in Fig. 8D, in which the MoS_2_ patch becomes increasingly distorted due to the introduction of an S-vacancy, as sulfur sharing is not possible.

From a thermodynamic perspective, the relative energy penalty associated with the formation of a sulfur vacancy was evaluated consistently within each supported system, as the energy difference between the interacting pristine and defective MoS_2_ systems normalized per MoS_2_ unit, as shown in Table S3.^47^ Despite the similar edge-mediated adsorption motifs observed for the pristine patch, sulfur-deficient MoS_2_ configurations show a higher penalty for the removal of a sulfur on ZnS (1.47 eV) than on ZrO_2_ (1.11 eV). This reflects the interfacial sharing occurring on the S-rich ZnS surface, which restores the coordination of MoS_2_ through covalent Mo–S and Zn–S bonds perturbing the ZnS lattice.

The analysis of the PDOS (Fig. 8E) confirms the interfacial reconstruction from an electronic structure perspective. The PDOS fingerprint of the sulfur atoms shared across the MoS_2−*x*_/ZnS interface are remarkably similar due to the substantial overlap of their areas. In fact, the full-energy range Tanimoto similarity coefficient (*T*_C_) was quantified as 0.410 (corresponding to an overlap fraction of ∼58%),^33^ in contrast to the dissimilarity between sulfur atoms in isolated MoS_2_ and ZnS (*T*_C_ = 0.006), reflecting intrinsically different electronic environments. Following the interfacial reconstruction, the sulfur atoms originating from MoS_2_ undergo the highest electronic reconstruction, as evidenced by a *T*_C_ value of 0.002 with respect to the same atoms in isolated MoS_2_. Conversely, the sulfur atoms originating from ZnS retain a significant degree of their electronic character (*T*_C_ = 0.48 with the corresponding atoms on isolated ZnS), while simultaneously rehybridizing at the interface. Overall, this indicates that the interfacial sulfur atoms exhibit a comparable hybridization and corroborates the formation of a shared sulfur electronic network at the MoS_2_/ZnS interface, primarily driven by the reorganization of MoS_2_, with ZnS acting as a sulfur-rich stabilizing environment that accommodates interfacial hybridization without fully losing its intrinsic electronic environment.

To further support this, the occupied centroids of the Mo 4d, S 3p and Zn 3d bands of MoS_2_/ZnS, MoS_2−*x*_/ZnS and ZnS were quantified. The changes in the states more involved in the interfacial bonding were highlighted by performing the analysis in proximity of the Fermi level, as shown in Fig. S16, focusing on the entire MoS_2_ patch and the outermost layer of the ZnS (unit formula: 12 Zn and 12 S atoms).^48,49^ In agreement with the PDOS similarity analysis, the sulfur chemical potential of both MoS_2_ and ZnS varied due to the reciprocal atoms sharing, as confirmed by the shift to higher energies of the S 2p states (from −0.95 to −0.72 eV for MoS_2_ and from −1.07 to −0.95 eV for ZnS).^49^ Accordingly, the Mo 3d band center of the patch shifts negatively (−0.51 eV to −0.75 eV), correlating with the increased Mo–S coordination,^50^ and the Zn 3d states of ZnS shifts positively (from −1.04 to −0.91 eV), correlating with the sulfur-sharing and in agreement with the structural changes in the Zn sub-lattice following the reorganization required to reconstruct the vacant MoS_2_ patch, shown in more detail in Fig. S17.^50^

The DFT-based simulation results suggest that the ZnS support interacts with MoS_2_*via* sharing of sulfur atoms while keeping the integrity of the 2H-MoS_2_ lattice. Such insights may explain the formation of few-layer MoS_2_ on ZnS verified experimentally, in contrast with the agglomeration of MoS_2_ observed at the surface of a ZrO_2_ support. In view of these results, the sharing of sulfur atoms may be also closely related to the increase in the Mo–S coordination number observed in the *in situ* XAS experiment, as the formation of additional S-vacancies in MoS_2_ may induce a bonding with sulfur from the MoS_2_/ZnS interface during the H_2_ pretreatment. While the dispersion of MoS_2_ in the form of few layers is expected to be a key factor leading to catalytic CO_2_ hydrogenation to methanol due to the likely presence of basal plane S-vacancies exposed at the surface, the potential role of the structural reconstruction verified during the H_2_ pretreatment on catalytic activity still deserves further investigation.

## Conclusions

In summary, this work demonstrates that MoS_2_ can be supported on ZnS by means of a facile wet impregnation method, leading to enhanced catalytic activity for CO_2_ hydrogenation to methanol in comparison with bulk MoS_2_. Catalyst characterization in combination with DFT-based simulation suggest that such an improvement may be associated with the sulfur atom sharing and structural similarity at the MoS_2_/ZnS interface, which help promote oriented growth of MoS_2_ in the form of few layers with basal plane S-vacancies exposed at the surface, as these defects have been previously identified as active sites for methanol production from CO_2_. In addition to this MoS_2_/ZnS interaction, *in situ* XAS and H_2_-TPR suggest that the H_2_ pretreatment induces further interplay between the sulfides, as S-vacancy formation in MoS_2_ promotes transfer of sulfur atoms from ZnS to MoS_2_, possibly mediated by H_2_S, thus leading to a partial reconstruction of the active phase. In contrast, when MoS_2_ is impregnated onto the reference ZrO_2_ support, the catalyst selectivity is considerably shifted towards methane, most likely in virtue of the more abundant edges in the MoS_2_ particles, which are in this case more agglomerated at the support surface. Given the key role of MoS_2_ morphology on CO_2_ hydrogenation activity, these findings emphasize the importance of structural similarity in MoS_2_-based composite catalysts, as this may be involved in the formation of a few-layer active phase on the ZnS support, and could also help explain other previous reports of a MoS_2_/ZnS synergistic mechanism.^14^

## Ulrich Schubert dedication

This work is dedicated to Professor Ulrich Schubert on the occasion of his 80th birthday, in honor of his outstanding contributions and inspiring mentorship.

## Author contributions

Conceptualization: G. A. S. A. and K. F. Methodology: G. A. S. A., S. B., A. T. and K. F. Formal analysis: G. A. S. A. and S. B. Investigation: G. A. S. A., T. W., A. T., C. E., M. S. P. and D. C. C. B. Writing – original draft preparation: G. A. S. A. and S. B. Writing – review & editing: G. A. S. A., S. B., K. F., D. C. C. B., A. T. Supervision: K. F. Funding acquisition: K. F.

## Conflicts of interest

There are no conflicts to declare.

## Supplementary Material

TA-014-D6TA01545J-s001

## Data Availability

The data supporting this article have been included as part of the supplementary information (SI). Supplementary information is available. See DOI: https://doi.org/10.1039/d6ta01545j.
