# Supplementary material for: Few-layer MoS2 dispersion induced by sulfur atom sharing to promote CO2 hydrogenation to methanol
Source: J Mater Chem A Mater. 2026 Jun 5;14(44):29662–71. doi: 10.1039/d6ta01545j (PMC13238494; doi:10.1039/d6ta01545j)
Supplement: TA-014-D6TA01545J-s001 [file TA-014-D6TA01545J-s001.pdf]

Supplementary Information (SI) for Journal of Materials Chemistry A.
This journal is © The Royal Society of Chemistry 2026

Supporting Information

Few-layer MoS_2 dispersion induced by sulfur atom sharing to promote CO_2 hydrogenation to methanol

Gustavo A.S. Alves[a], Silvio Bellomi[a], Tobias Wagner[a], Alberto Tampieri[a], Christian Etzlstorfer[a], Michael Stöger-Pollach[b], Daniel C. Cano-Blanco[c,d], Karin Föttinger[a*]

[a] *Institute of Materials Chemistry, TU Wien, Getreidemarkt 9/BC/01, 1060 Vienna, Austria*

[b] *University Service Center for Transmission Electron Microscopy (USTEM), TU Wien, Stadionallee 2/057-02, 1020 Vienna, Austria*

[c] *Paul Scherrer Institute, PSI Center for Energy and Environmental Sciences, Villigen PSI, CH-5232, Switzerland*

[d] *École Polytechnique Fédérale de Lausanne (EPFL), Institute of Chemical and Bioengineering, Lausanne CH-1015, Switzerland*

* Corresponding author

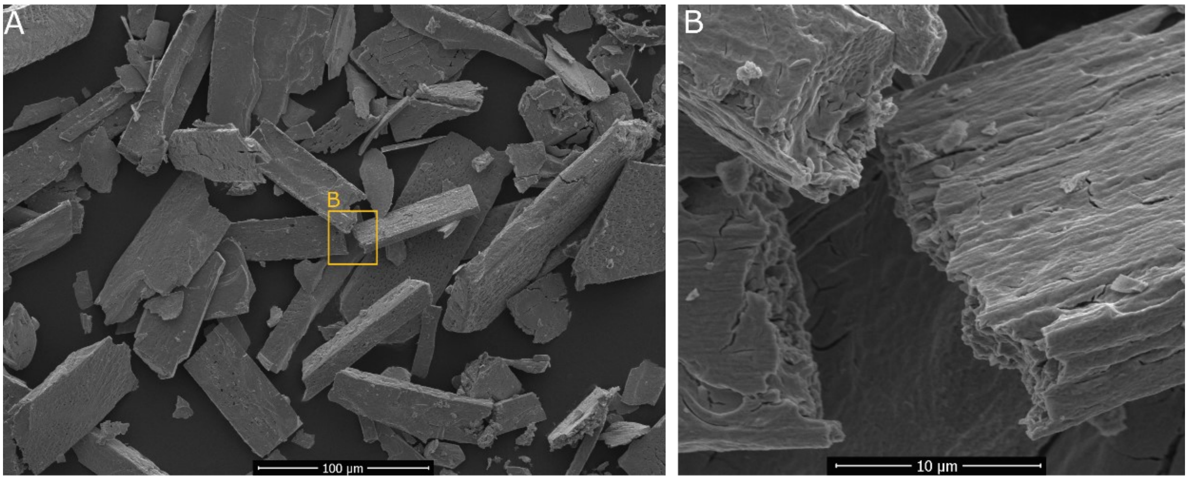

Figure S1 - SEM micrographs of bulk MoS_2 produced by thermal treatment of ATTM, under 1000x (A) and 10000k (B) magnification

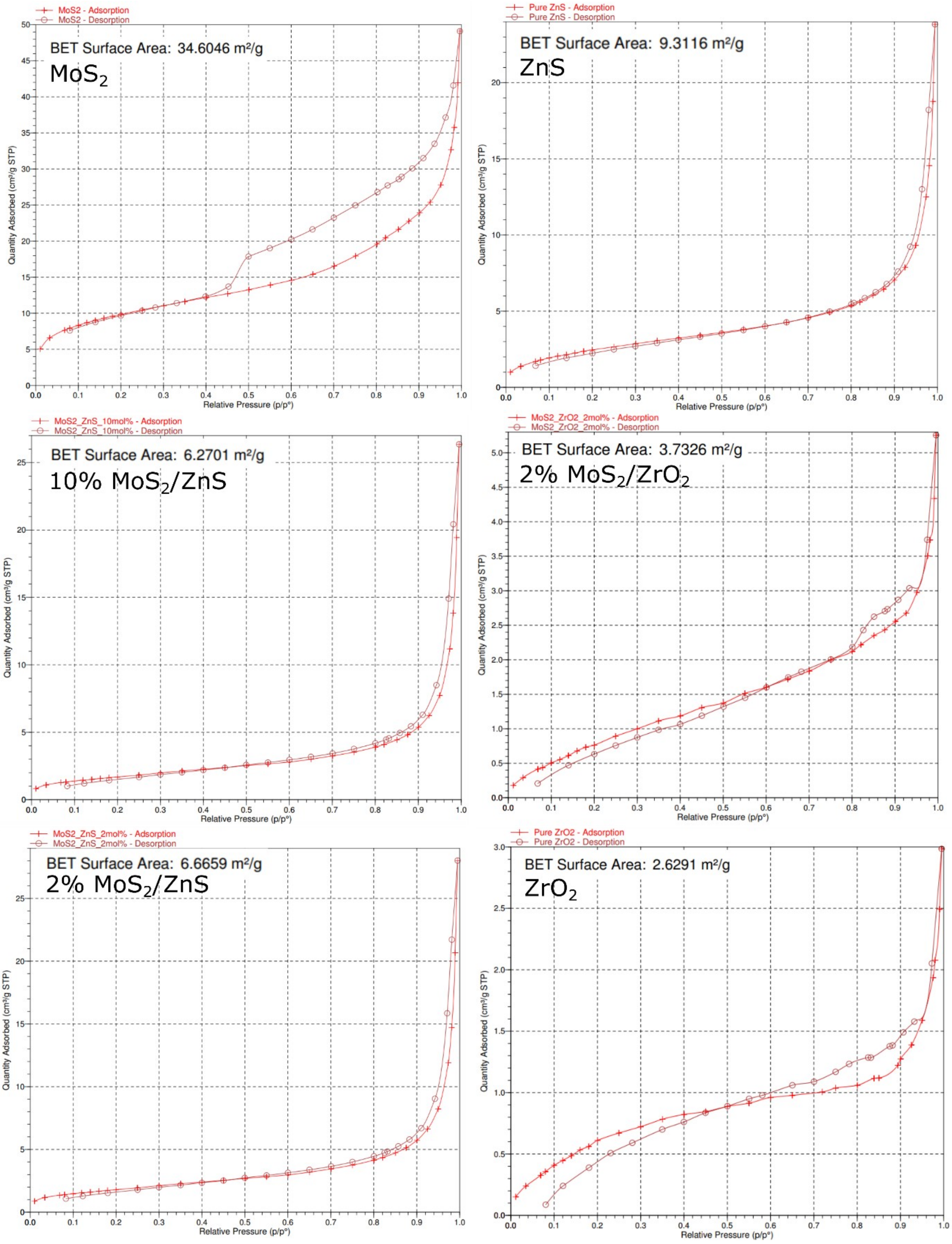

Figure S2 – N_2 adsorption-desorption isotherms and surface areas determined by the BET method for as-synthesized MoS_2, ZnS, ZrO_2 2% MoS_2/ZnS, 10% MoS_2/ZnS and 2% MoS_2/ZrO_2

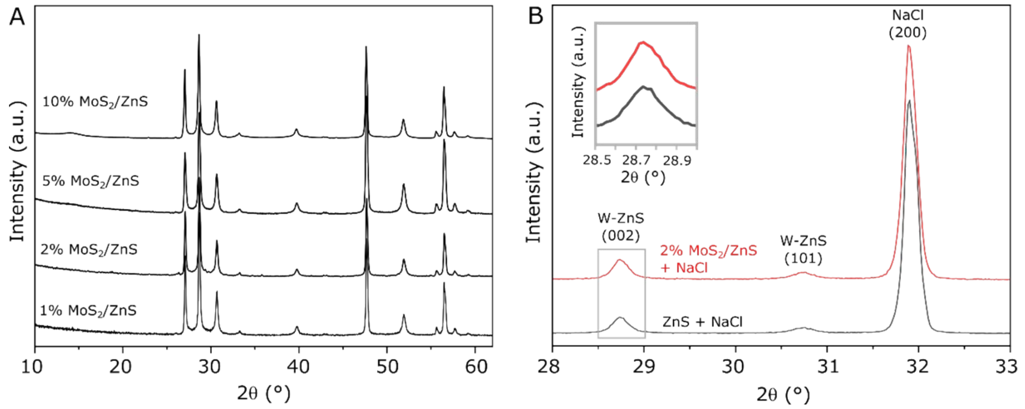

Figure S3 - X-ray diffraction patterns of 10, 5, 2 and 1% MoS_2/ZnS (A) and comparison of 1% 2% MoS_2/ZnS with ZnS using NaCl as an internal standard

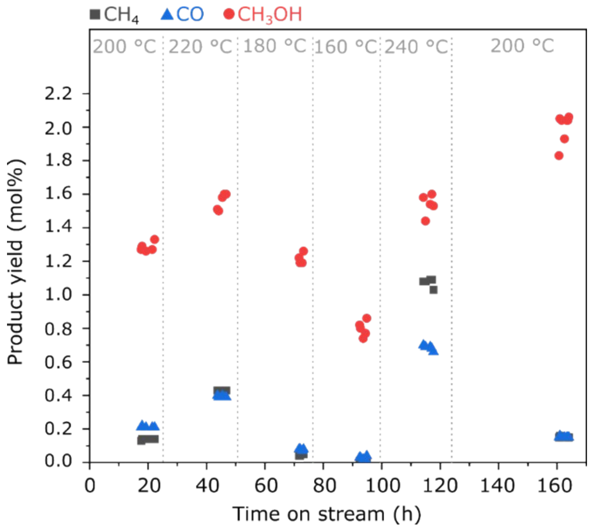

Figure S4 - Catalytic activity of 2% MoS_2/ZnS in a fixed-bed reactor at 20 bar/200 °C/0.5 g_{cat} in terms of CH_4, CO and CH_3OH yields at different reaction temperatures

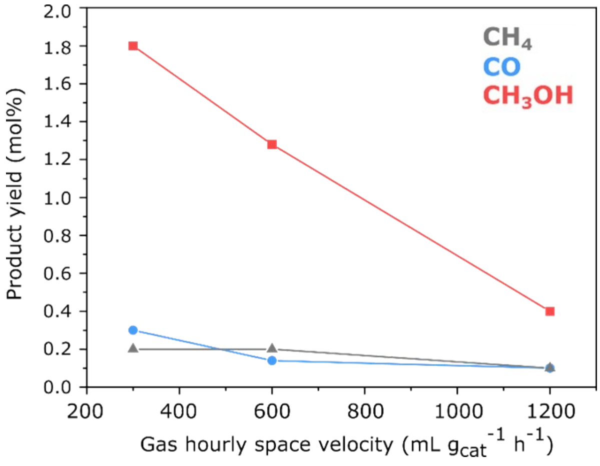

Figure S5 - Catalytic activity of 2% MoS_2/ZnS in a fixed-bed reactor at 20 bar/200 °C in terms of CH_4, CO and CH_3OH yields using 1, 0.5 or 0.25 g_{cat}.

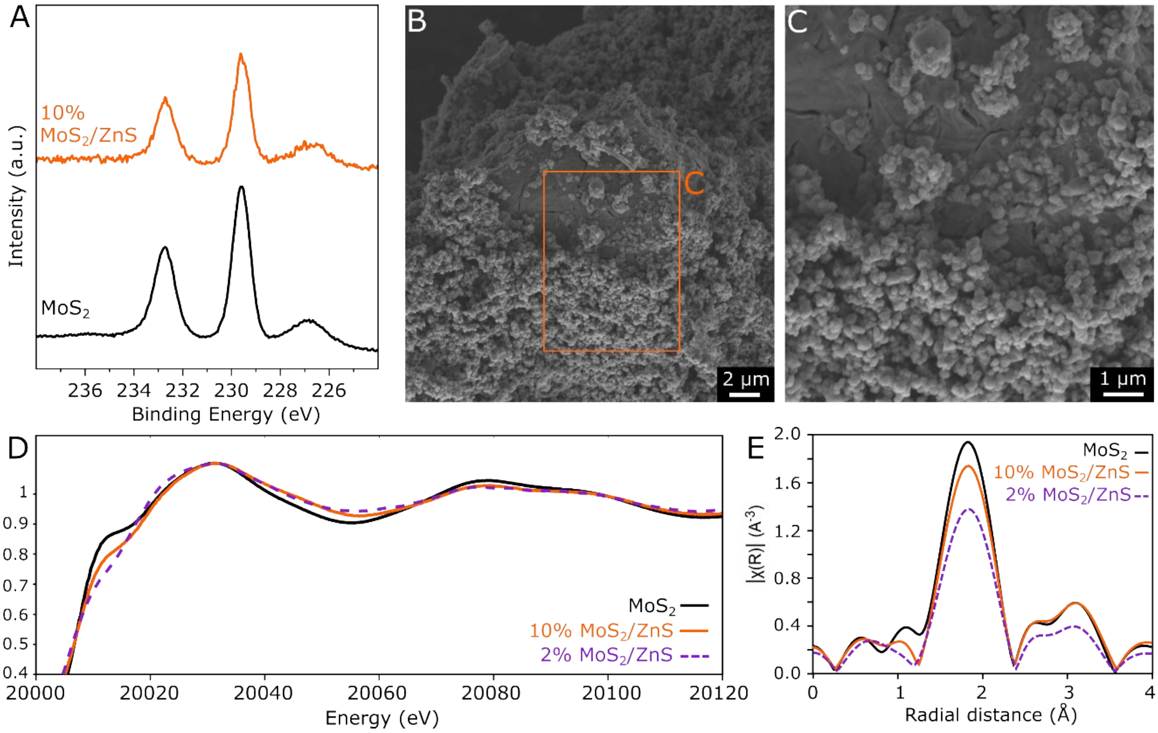

Figure S6 - High-resolution XPS spectrum of the Mo 3d/S 2s region (A), SEM micrographs (B,C), Mo K-edge XANES (D) and EXAFS (E) spectra of as-synthesized 10% MoS_2/ZnS compared with relevant samples

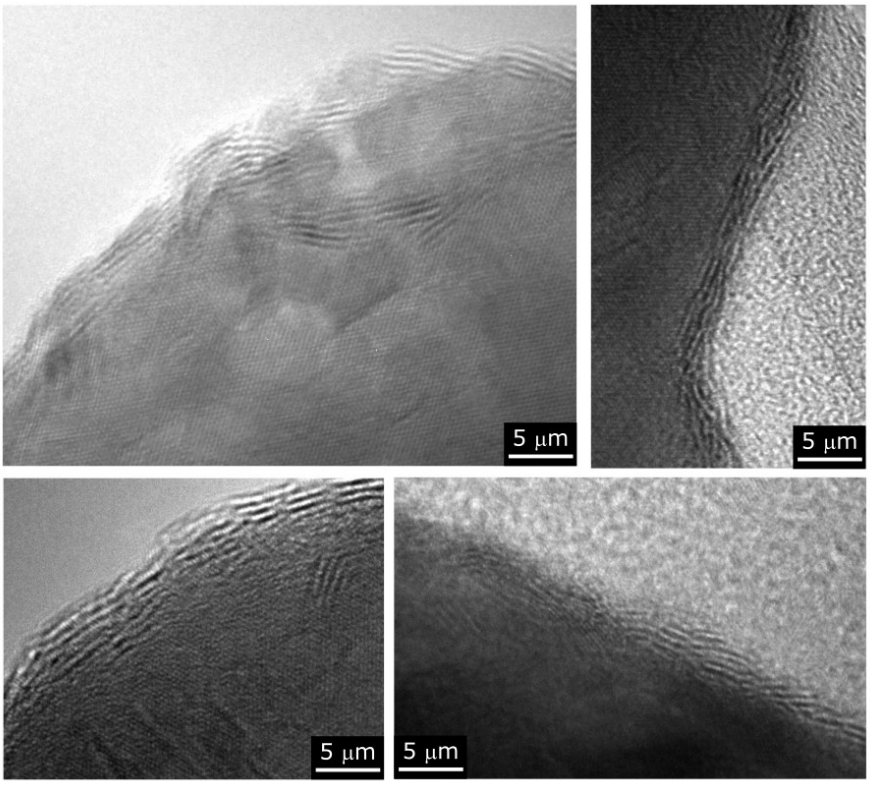

Figure S7 - Additional Transmission Electron Microscopy images of 2% MoS_2/ZnS

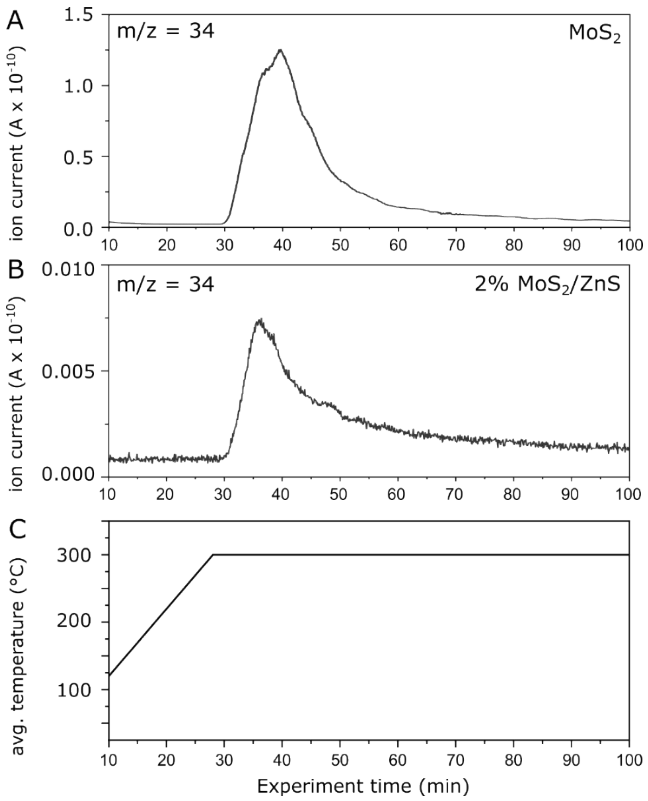

Figure S8 - Evolution of H_2S (m/z = 34) from MoS_2 (A) and 2% MoS_2/ZnS (B) measured by mass spectrometry during H_2 treatment at 300°C following a 10 °C/min heating ramp (C)

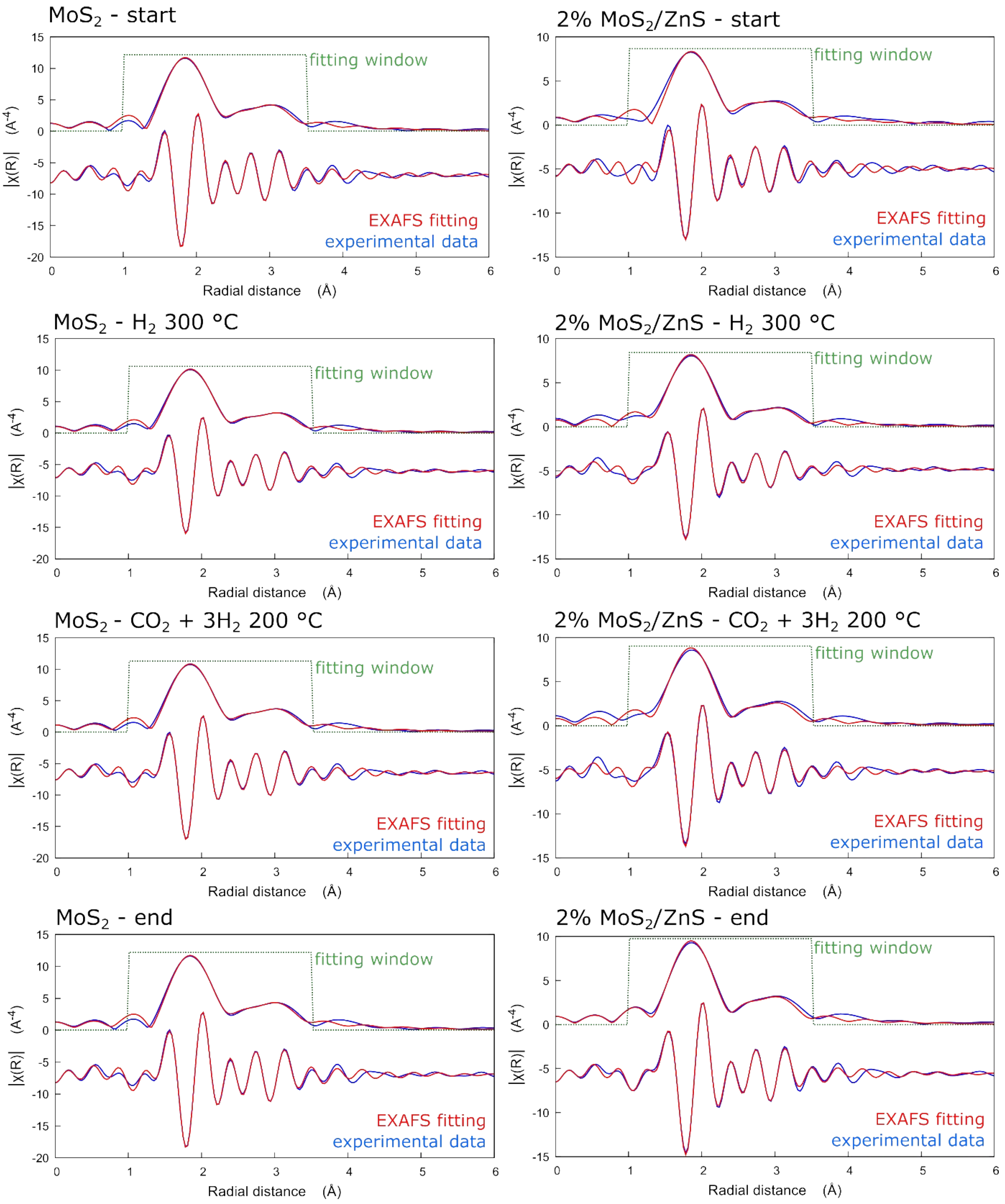

Figure S9 - Summary of EXAFS fitting compared with *in-situ* experimental data for MoS_2 and 2% MoS_2/ZnS

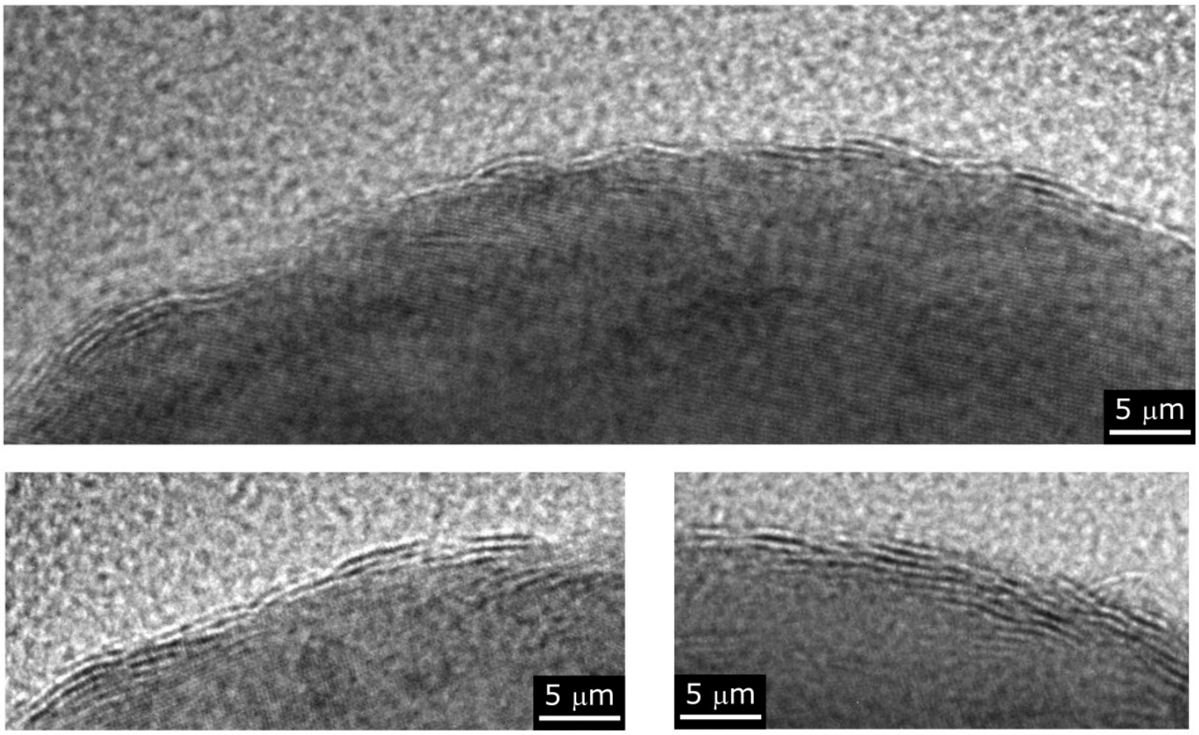

Figure S10 - Transmission Electron Microscopy images of 2% MoS_2/ZnS after H_2 treatment at 300°C

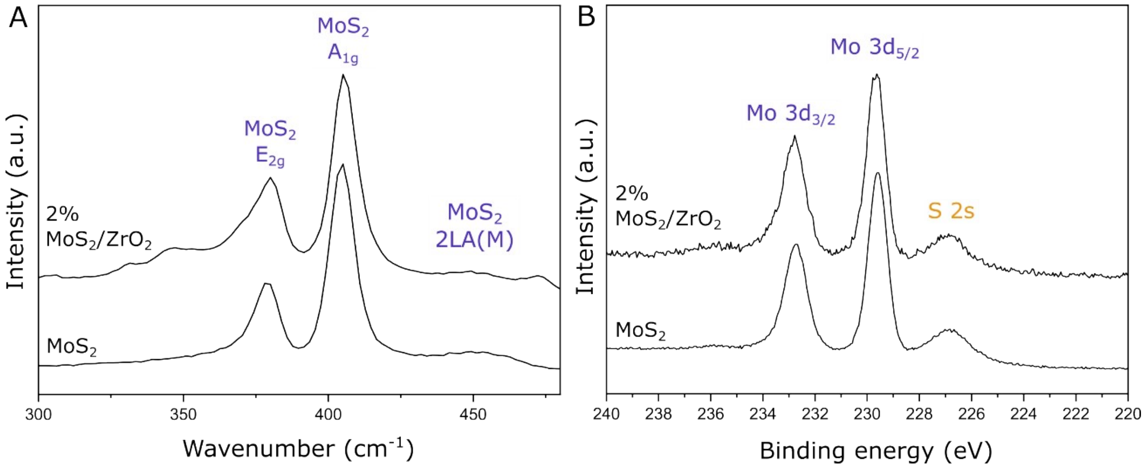

Figure S11 - Raman spectrum with 532 nm excitation (A) and high-resolution XPS spectrum of the Mo 3d/S2s region (B) of pure MoS_2 and 2% MoS_2/ZrO_2

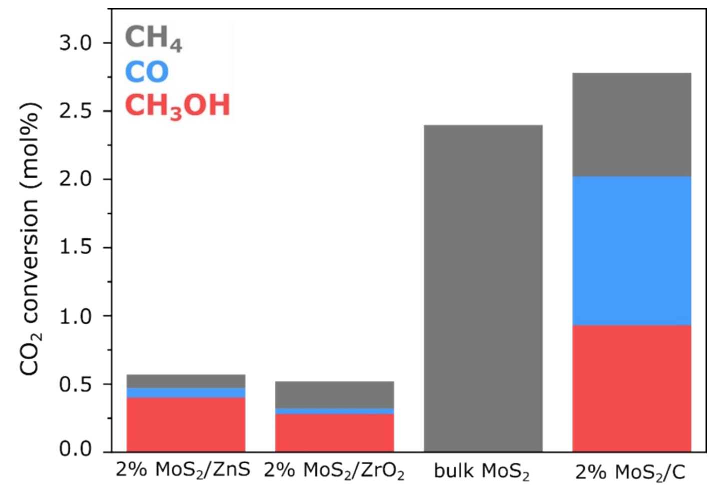

Figure S12 - Catalytic activity of 2% MoS_2/C at 20 bar/200 °C/0.25 g_{cat} compared with MoS_2, 2% MoS_2/ZnS and 2% MoS_2/ZrO_2 in terms of CO_2 conversion to CH_4, CO and CH_3OH

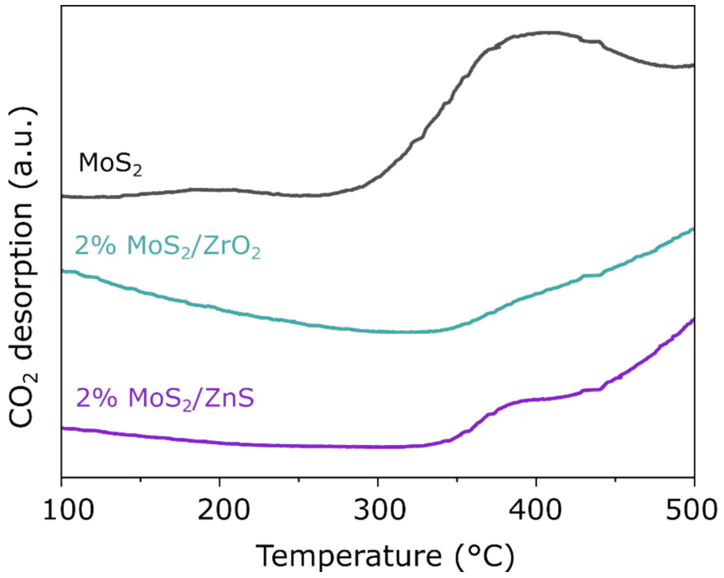

Figure S13 – CO_2-TPD profiles of MoS_2, 2% MoS_2/ZnS and 2% MoS_2/ZrO_2

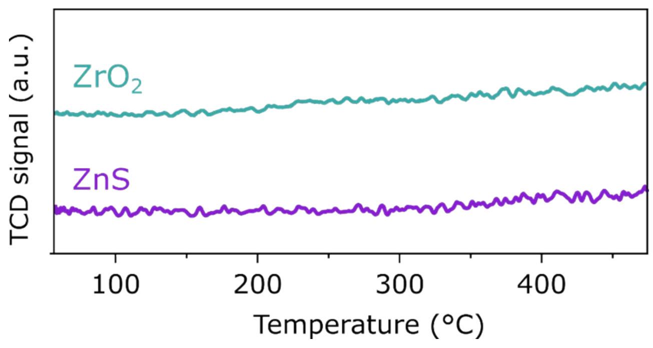

Figure S14 – H_2-TPR profile of the ZnS ZrO_2 supports

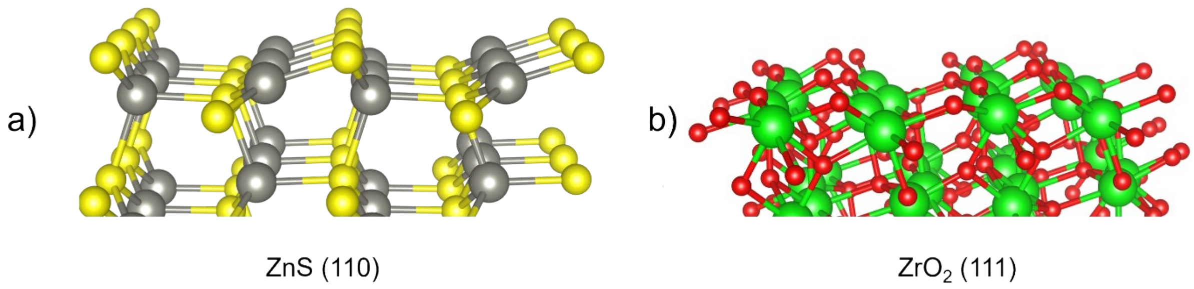

Figure S15 - Optimized structural models for the ZnS (100) and ZrO_2 (111) terminations

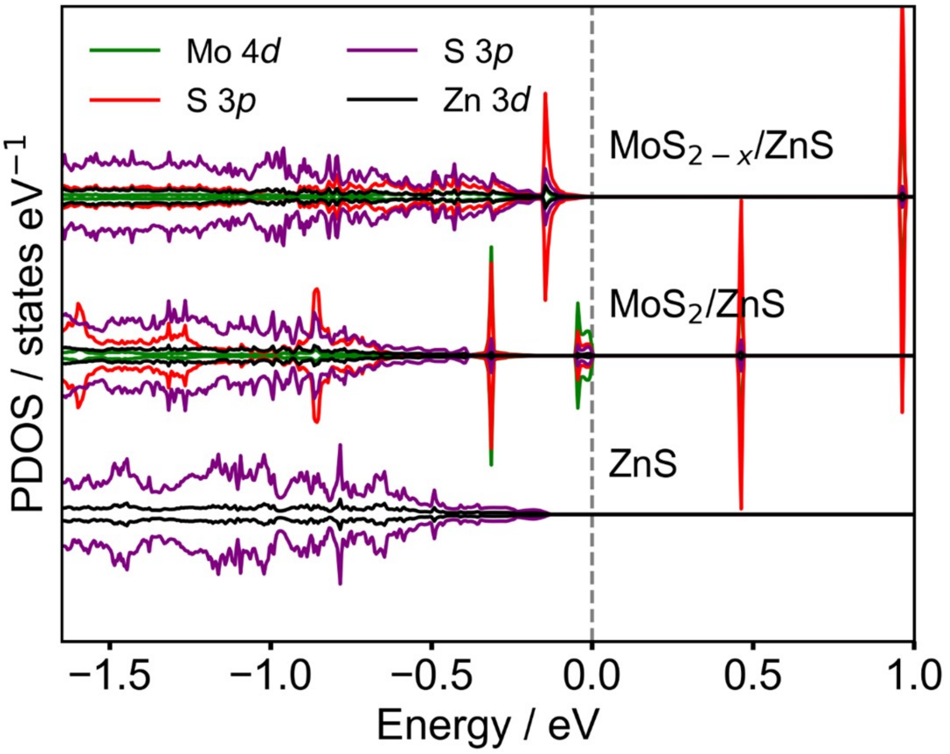

Figure S16 - Partial density of states in proximity of the Fermi level used to calculate the occupied centroids of the Mo 4*d*, S 3*p* and Zn 3*d* states for MoS_{2-x}/ZnS, MoS_2/ZnS and ZnS.

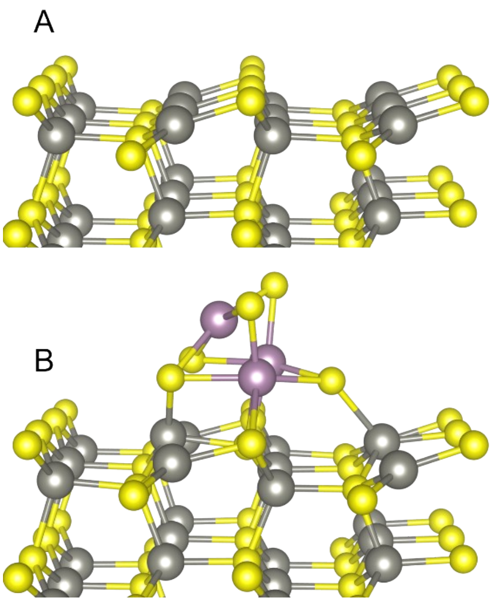

Figure S17 - Optimized structures of ZnS and MoS_{2-x}/ZnS demonstrating the reorganization of the Zn and S sub-lattices of ZnS required to accommodate the vacant MoS_{2-x} patch.

**Model**	**Supercell**	**In-plane dimensions (Å)**	**γ cell angle (°)**
MoS_2 monolayer	4×4×1	12.6×10.9	120
ZnS (100)	3×2×1	11.4×12.5	90
ZrO_2 (111)	2×2×1	14.6x14.8	114.7

Table S1 – Characteristics in-plane dimensions of the supercells for the different terminations.

		**N (Mo-S)**	**N (Mo-Mo)**	**σ² (Å²)**	**ΔE (Mo-S) (eV)**	**ΔE (Mo-Mo) (eV)**
MoS_2	Start	5.6 ± 0.7	3.4 ± 1.0	0.003 ± 0.002	1.4	0.5
MoS_2	H_2 300 °C	5.5 ± 0.6	3.0 ± 0.8	0.005 ± 0.001	0.7	0.7
MoS_2	CO_2 + H_2 300 °C	5.6 ± 0.7	3.3 ± 0.9	0.004 ± 0.001	1.4	0.4
MoS_2	End	5.6 ± 0.7	3.5 ± 0.9	0.003 ± 0.001	1.3	0.7
2% MoS_2/ZnS	Start	3.7 ± 1.1	2.0 ± 1.4	0.003 ± 0.003	3.3	- 2
2% MoS_2/ZnS	H_2 300 °C	5.7 ± 0.6	2.9 ± 0.9	0.008 ± 0.001	2.8	- 0.3
2% MoS_2/ZnS	CO_2 + H_2 300 °C	5.9 ± 0.8	3.4 ± 1.2	0.007 ± 0.002	3.3	0.5
2% MoS_2/ZnS	End	5.8 ± 0.7	3.7 ± 1.0	0.006 ± 0.001	3.0	0.2

Table S2 - Summary of EXAFS fitting parameters for the *in situ* XAS experiment

Structure	E (eV)	ΔE(MoS_{2-x}-MoS_2)/N(MoS_2)* (eV)
MoS_2/ ZnS	-488.57	-
MoS_{2-x}/ZnS	-484.18	1.47
MoS_2/ ZrO_2	-1877.65	-
MoS_{2-x}/ ZrO_2	-1874.33	1.11
		*N(MoS_2) = 3

Table S3 - Total electronic energy values (eV) for the optimized interfaces and energy difference between the pristine and defective interface normalized by the number of MoS_2 unit formula.
